# Aspirin Bioactivity for Prevention of Cardiovascular Injury in COVID-19

**DOI:** 10.3389/fcvm.2020.562708

**Published:** 2020-11-30

**Authors:** Temistocles Diaz, Barry H. Trachtenberg, Samuel J. K. Abraham, Rao KosagiSharaf, Armando A. Durant-Archibold

**Affiliations:** ^1^Internal Medicine-Interventional Cardiology, Department of Cardiology, Punta Pacifica Hospital, Affiliate of John Hopkins Medicine International, Panama City, Panama; ^2^Biomedicine Research Unit, Center for Biodiversity and Drug Discovery, Instituto de Investigaciones Científicas y Servicios de Alta Tecnología (INDICASAT Asociation of Public Interest), Panama City, Panama; ^3^Department of Cardiology, Houston Methodist DeBakey Heart and Vascular Center, Houston, TX, United States; ^4^Department of Surgery & Centre for Advancing Clinical Research (CACR), Yamanashi University, Chuo, Japan; ^5^Edogawa Evolutionary Laboratory of Science (EELS), Edogawa Hospital, Tokyo, Japan; ^6^The Mary-Yoshio Translational Hexagon (MYTH), Nichi-In Centre for Regenerative Medicine (NCRM), Chennai, India; ^7^Department of Biochemistry, College of Natural, Exact Science and Technology, Universidad de Panama, Panama City, Panama

**Keywords:** molecular mechanisms, aspirin, SARS-CoV-2, COVID-19, prevention of cardiovascular disease

## Introduction

The coronavirus disease 2019 (COVID-19), caused by the severe acute respiratory syndrome coronavirus 2 (SARS-CoV-2), has come to be one of the gravest pandemics of the last two centuries. WHO epidemiological records on COVID-19 outbreak confirmed more than 35 million cases and 1 million deaths worldwide since the disease have originated in Wuhan, China (1, 2). Although clinical treatment of COVID-19 patients focuses on the pulmonary complications and acute respiratory distress syndrome (ARDS), medical reports have also pointed toward the severe deterioration of the patient's state of health due to cardiovascular complications. Furthermore, cardiovascular comorbidities have been determined as key factors of mortality for SARS-CoV-2 infected patients, which present high blood levels of cardiac-specific proteins troponin I and/or T, indicative signs of hypoxia, tachyarrhythmia, myocarditis, and myocardial injury (3–8). Other cardiovascular injuries associated with COVID-19 are venous and arterial thrombosis, and venous thromboembolism (VTE). Case studies performed on COVID-19 patients, and autopsies conducted on those who died due to cardiovascular complications such as stroke and acute coronary syndromes, point to thrombotic disease as a critical factor of mortality in severe cases of COVID-19 (9–12).

Severe cases of SARS-COV-2 infected patients experience lymphocytopenia and a high activation of metabolic proinflammatory cytokines mechanisms which leads to an elevated blood concentration of interleukin (IL) 2 (IL-2), IL-6, IL-7, interferon gamma (IFN-γ), macrophage inflammatory protein- 1 alpha (MIP1A), and tumor necrosis factor alpha (TNF-α) pro-inflammatory cytokines (4, 13). This high level of cytokines, known as cytokine storm syndrome (CSS), tends to be a critical factor of morbidity and mortality for COVID-19 patients. CSS contributes to the upregulation of metabolic coagulation pathways resulting in damage to the endothelium, and therefore to the cardiovascular system (14–16). Furthermore, oxygen deprivation seems to mediate the hypercoagulability in COVID-19 (9). Patients with severe COVID-19 pneumonia progress to ARDS, accompanied by disseminated intravascular coagulation (DIC) (17), which may upregulate the coagulation pathways by activation of procoagulant factors, such as tissue factor, leading to both arterial and venous thrombotic disease. These biochemical mechanisms are main factors associated with disturbance of blood coagulation in COVID-19 patients. On the other hand, there is a potential risk of VTE associated with administration of some medications to severe cases of COVID-19 (18). Clinical reports have indicated thrombotic disease in around 25–30% SARS-CoV-2 infected patients, mainly in seriously ill patients (9, 10, 12, 19–22). Therefore, early anticoagulant treatment certainly leads to a better prognosis. In this sense, the antithrombotic properties of aspirin make it a plausible drug for thrombotic disease prevention, the efficacy of which requires to be validated in COVID-19 patients.

**Figure 1:**
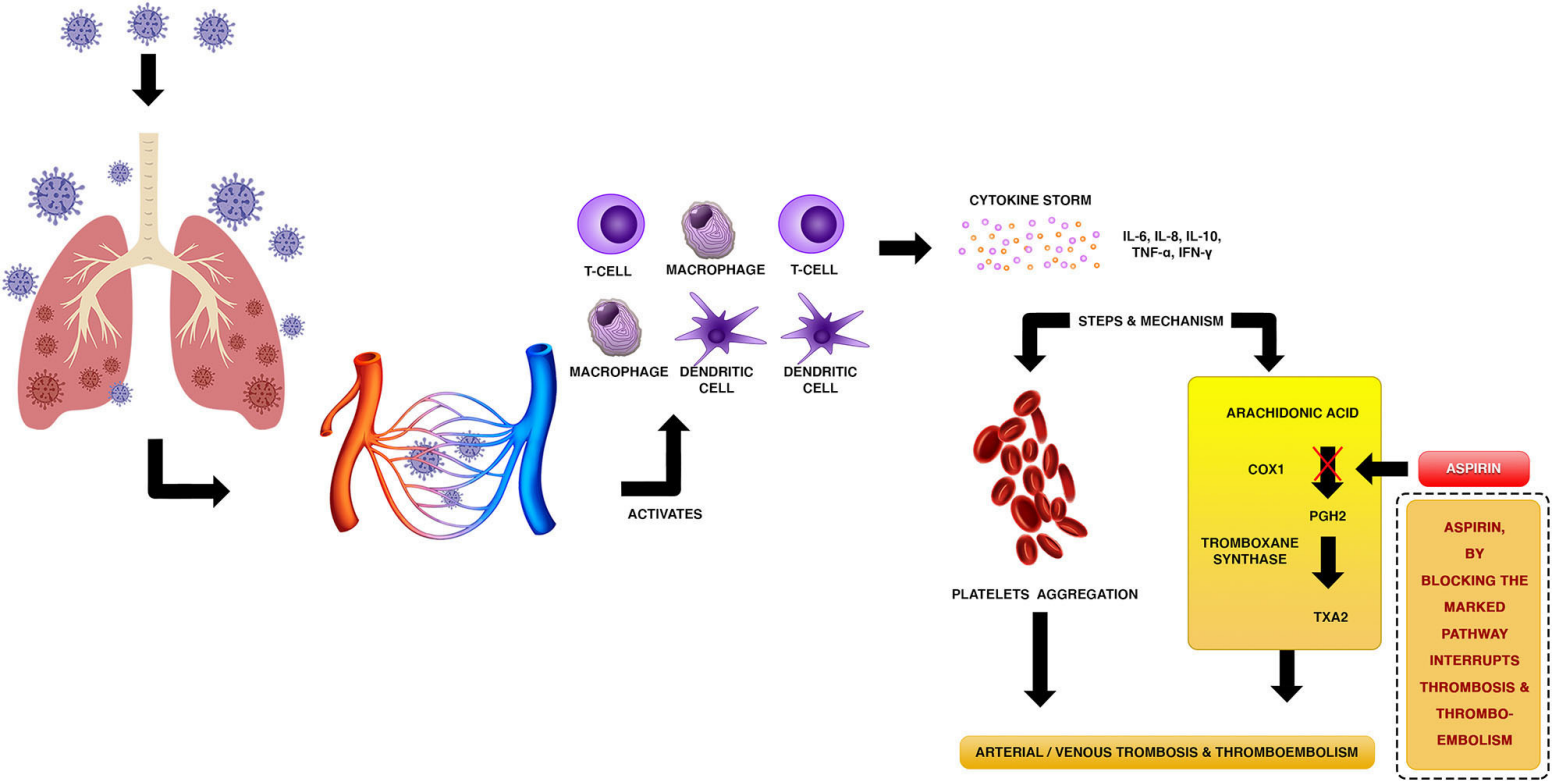
Putative mechanism of thrombotic disease associated with cytokine storms in COVID-19 patients, and biochemical mechanism of aspirin to prevent arterial/venous thrombosis and thromboembolism.

Acetylsalicylic acid, aspirin, is an antiplatelet drug that inhibits platelet aggregation. The main biochemical mechanism by which aspirin inhibits thrombotic damage is through irreversible inactivation of cyclooxygenase 1 (COX-1) enzyme. In this respect, aspirin acetyl group attaches to the active side of COX-1 at S529, inhibiting the biosynthesis of prostaglandin H2 (PGH2), which is the substrate of thromboxane-A synthase that catalyzes the generation of the prothrombotic eicosanoid thromboxane A2 (TXA2) (Figure 1) suppressing platelet aggregation leading to the prevention of VTE without significant alterations in the endothelial function. On the other hand, aspirin acetylates fibrinogen and other proteins involved in blood coagulation, also preventing thrombus formation. These biochemical mechanisms cause a decrease of dense granule release from platelets (23, 24). Activated platelets and endothelial cells biosynthesize P-selectin, a cell-adhesion glycoprotein that promotes leukocyte and platelet adhesion, and the attachment of leukocytes to the vascular endothelium. Aspirin inhibits P-selectin, which results in the reduction of deep vein thrombosis (DVT) (25). Other antithrombotic mechanism of aspirin involves upregulation of nitric oxide (NO) metabolic production by endothelial cells, through inhibition prostacyclin synthesis, which leads to platelet inactivation (26). Additionally, aspirin prevents the formation of the serine protease enzyme thrombin, which catalyzes the transformation of fibrinogen to fibrin and, hence, leads to the formation of blood clot. Furthermore, thrombin is a potent mediator of platelet activation and aggregation. Thrombin promotes its biosynthesis by feedback activation of prothrombinase complex coenzymes (factors V and VIII). Aspirin inhibits tissue factor (TF): Factor VIIa (FVIIa) complex that catalyzes thrombin formation and, therefore, thrombin-mediated coagulation pathways (24).

Aspirin is certainly one of the most used drugs in medicine. In addition to its antithrombotic activity, aspirin is very well-known for its antipyretic, antiviral, and analgesic properties (27). Aspirin bioactivity inhibits virus replication such as influenza virus, hepatitis C virus, and flavivirus. Among the reported metabolic mechanisms that produces inhibition is the activation of mitogen-activated protein kinase/extracellular signal-regulated kinase kinase ½ and p38 mitogen-activated protein kinase. Furthermore, an additional mechanism is the inhibition of the proinflammatory transcription factor NF-κB which is relevant for viral genes expression (28). Clinical studies have reported reduction in the rate of stroke, peripheral artery disease, thromboembolism, and myocardial infarction (MI). At present, aspirin is recommended for primary and secondary prevention of stroke, anterior MI with left ventricular thrombus, peripheral artery disease, and acute coronary syndrome (29, 30). A multicenter, double-blind, placebo-controlled study WARFASA (the Aspirin for the Prevention of Recurrent Venous Thromboembolism Warfarin and Aspirin) for the assessment of the efficacy of aspirin for prevention and treatment of VTE, which included 403 patients, showed a lower VTE in patients who received aspirin than in patients not receiving antithrombotic treatment (28 [6.6%] of 205 patients vs. 43 [11.2%] of 197 patients) (31). Furthermore, in a more recent clinical trial of 1,224 patients on the prevention of recurrent unprovoked venous thromboembolism by aspirin, performed by the International Collaboration of Aspirin Trials for Recurrent Venous Thromboembolism (INSPIRE), it was found that aspirin reduces the risk of recurrence of DVT by 34%, without significantly increasing the risk of bleeding (32). Clinical studies from China showed that ARDS developed in a short period of time in COVID-19 patients, which leads to a high number of deaths in severe cases of the disease (74%) (33, 34). ARDS causes uncontrolled coagulation disfunction in severely ill patients (35). Clinical trials reported a decrease in number of cases of ARDS in patients treated with aspirin, which is can be explained by the antithrombotic properties of the drug (36). On the other hand, there are an important number of clinical investigations, registered at ClinicalTrials.gov, which are currently studying the bioactivities of aspirin in COVID-19 patients (NCT04365309; NCT04363840; NCT04333407; NCT04343001; NCT04324463; NCT04368377; NCT04410328; NCT04425863; NCT04466670; NCT04498273) (37).

In the light of the evidence discussed in this article, it is clear that patients infected with SARS-CoV-2 virus experience an increment of proinflammatory cytokine molecules, which is a main factor that leads to abnormal platelet aggregation, which causes thrombosis and thromboembolism in COVID-19 patients. Thrombotic disease leads to alterations of many organs, mainly the lung, and the cardiovascular system. Treatment of patients in early stages of COVID-19 with low-dose aspirin (75–100 mg) represents an important pharmacological strategy for prevention of platelet aggregation, which leads to a predictable potential disease progression of arterial/venous thrombosis and thromboembolism, based on the metabolic mechanisms of action of this drug. The incorporation of aspirin therapeutical evaluation into clinical trials that studies the effectiveness of the drug on early stages patients with COVID-19, as well as its concomitant use with antiviral drugs, will enhance our knowledge regarding the physiological factors which promotes health and the development of more accurate therapeutical protocols.

## Author Contributions

RK and AD-A started the debate topic. TD, BT, SA, RK, and AD-A participated in discussions and made substantial contributions to the conception of the work, and writing of the manuscript. All authors read and agreed to its submission. All authors contributed to the article and approved the submitted version.

## Conflict of Interest

The authors declare that the research was conducted in the absence of any commercial or financial relationships that could be construed as a potential conflict of interest.
